# Demographic comparison and population projection of *Rhynchophorus ferrugineus* (Coleoptera: Curculionidae) reared on sugarcane at different temperatures

**DOI:** 10.1038/srep31659

**Published:** 2016-08-22

**Authors:** Lu Peng, Yunxin Miao, Youming Hou

**Affiliations:** 1 Key Laboratory of Insect Ecology in Fujian, College of Plant Protection, Fujian Agriculture and Forestry University, Fuzhou 350002, Fujian China; 2 Key Laboratory of Integrated Pest Management for Fujian-Taiwan Crops, Ministry of Agriculture, Fuzhou 350002, Fujian, China

## Abstract

Understanding how temperature affects fitness is important for conservation and pest management, especially in the era of global climate change. *Rhynchophorus ferrugineus* (Oliver) (Coleoptera: Curculionidae) is a worldwide pest of many economically important crops. Although much is known about this pest’s life cycle, its adaptability to different temperatures is not fully understood. Here, we used age- and stage-specific life tables to investigate the effects of temperature on fitness-related traits and demographic parameters of *R. ferrugineus* under eight constant temperature regimens in the laboratory. The growth potential of these populations was also evaluated. The greatest longevity for males and females was 158.0 d at 24 °C and 144.5 d at 21 °C, respectively, but mean total fecundity was the highest at 27 °C. The intrinsic rate of increase (*r*), finite rate of increase (λ), and net reproductive rate (*R*_0_) increased initially at low temperatures and then decreased. All metrics reached a maximum at 27 °C and a minimum at 36 °C. Mean generation times (*T* ) decreased across the temperature range with a minimum at 36 °C. Our results indicate that the optimum temperature for growth of *R. ferrugineus* was approximately 27 °C. Our work will be of value for developing strategies for control management of this pest species.

The red palm weevil, *Rhynchophorus ferrugineus* (Olivier) (Coleoptera: Curculionidae), is an extremely invasive and primary pest of the palm family. It attacks more than 20 palm species belonging to 16 different genera^1^. *R. ferrugineus* originated from South and Southeast Asia but has now spread to most palm-growing regions in Asia, Africa, Europe, and Oceania^2^. In China, the first reported infestation was identified in *Cocos nucifera* in Guangdong province in 1997 3. Subsequently, owing to transportation of infested plants, *R. ferrugineus* invaded and caused serious damage in many areas of China, including Hainan, Guangdong, Guangxi, Yunnan, Fujian, Hong Kong, Taiwan, Zhejiang, Jiangxi, and Shanghai^4^. Since *R. ferrugineus* larvae feed within the palm trunks and the resulting damage is generally only visible after long-term infection and considerable damage has occurred^5^, early detection is usually difficult. In addition, this behaviour provides the larvae with considerable protection against chemical insecticides, natural enemies, and pathogens. As a result, there is considerable current interest in developing an integrated pest management strategy based on pheromone traps and biological control^6^. Hence, knowledge of the life history and ecological adaptability of *R. ferrugineus* is important for identifying the optimal times for intervention to achieve more effective control management.

Environmental temperature is a significant factor influencing behaviour, distribution, development, survival, and reproduction in ectothermic organisms such as insects^7^,^8^. Knowledge of the temperature-dependent population growth potential of insect pest species is essential for predicting potential changes in population dynamics and for implementing efficient, economic, and ecological pest control strategies, especially in the context of predicted global climate warming^9^.

Analysis of the life history of *R. ferrugineus* will provide the data for predicting population peaks, establishing the timing for sampling operations and ecological zoning^10^, and developing integrated control programmes^11^. To date, the thermal requirements and the lower temperature thresholds for development, oviposition, and egg hatching in *R. ferrugineus* have been described^5^,^12^ along with effects on emergence^13^ and population growth^14^. However, there is limited information on how temperature affects population demographics and age- and stage-specific traits, which are important for metamorphic insects^15^. The traditional female age-specific life table ignores some important factors such as male individuals and stage differentiation, which may result in some problems. As both males and females are economically important and affect population dynamics, it is important to have information on both sexes. In addition, variations in developmental rates among individuals may help a population survive in unpredictable and harsh environmental conditions^16^.

The age- and stage-specific life table approach is a useful tool for conservation and pest management^17^, and is commonly employed to determine the growth parameters and the maximal growth potential of populations of insect pests^18^,^19^,^20^,^21^, mites^17^, and predators^22^ under different environmental conditions. This study was designed to quantify the manifold effects of temperature on the population fitness of *R. ferrugineus*. We used the age-stage, two-sex life table approach^23^ to investigate life-history traits, and to evaluate their impacts on the population demography of *R. ferrugineus* under different temperatures. Furthermore, the population growth potential of the weevil was estimated. This is the first comprehensive study of the effects of temperature on *R. ferrugineus* populations based on age stage-specific traits^15^. The results of our study showed that life history traits and demographic parameters were altered by changes in environmental temperatures; moreover, these effects were developmental stage- and age-specific. Our findings offer valuable insights into the establishment potential of *R. ferrugineus* in new environments with diverse temperature regimens and will be of value for the management of this pest species.

## Results

### Age stage-specific survival rate

**Figure 1:**
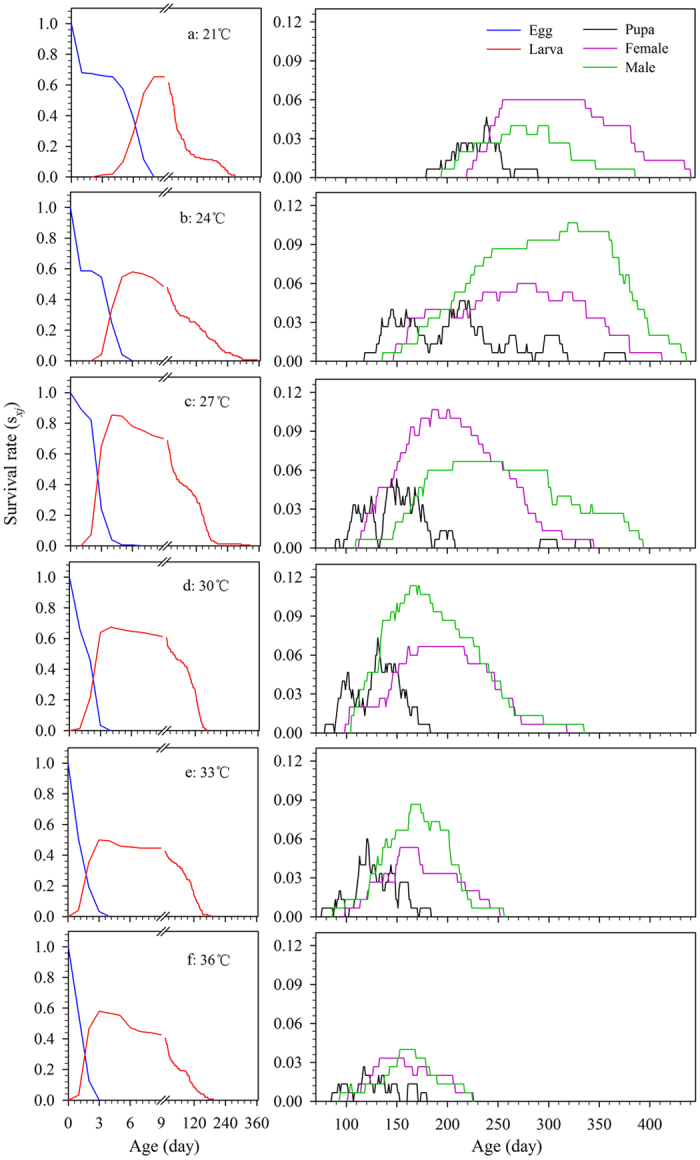
Age-stage specific survival rates (*s*_*xj*_) of *R. ferrugineus* reared on sugarcane at different constant temperatures. *s*_*xj*_, the probability that a newly laid egg will survive to age *x* and stage *j*.

At 15 and 18 °C, *R. ferrugineus* failed to complete development and reproduction. Data from these two temperatures were therefore excluded from all analyses. The age-stage survival curve, *s*_*xj*_, depicts the probability that a newly laid egg will survive to age *x* and stage *j* (Fig. 1a–f). The overlaps between different stages occur as a result of inter-individual variation in development rates. We found that the curve for survival of larvae had the slowest increase at 21 °C until the 10th day with a maximum survival rate of 65.3% (Fig. 1a). The fastest increasing curves were seen at 33 and 36 °C, but reach to the maximal survival rates of only 50% and 58%, respectively (Fig. 1e,f). At 27 °C, the curve took 4 d (one day more than at 33 and 36 °C) to reach the maximum survival rate of 85.3% (Fig. 1c); this survival rate was greater than at other temperatures. In addition, the curves of female and male emerged earliest at 33 °C, with 99 and 87 d, respectively (Fig. 1e).

### Age stage-specific fecundity

**Figure 2:**
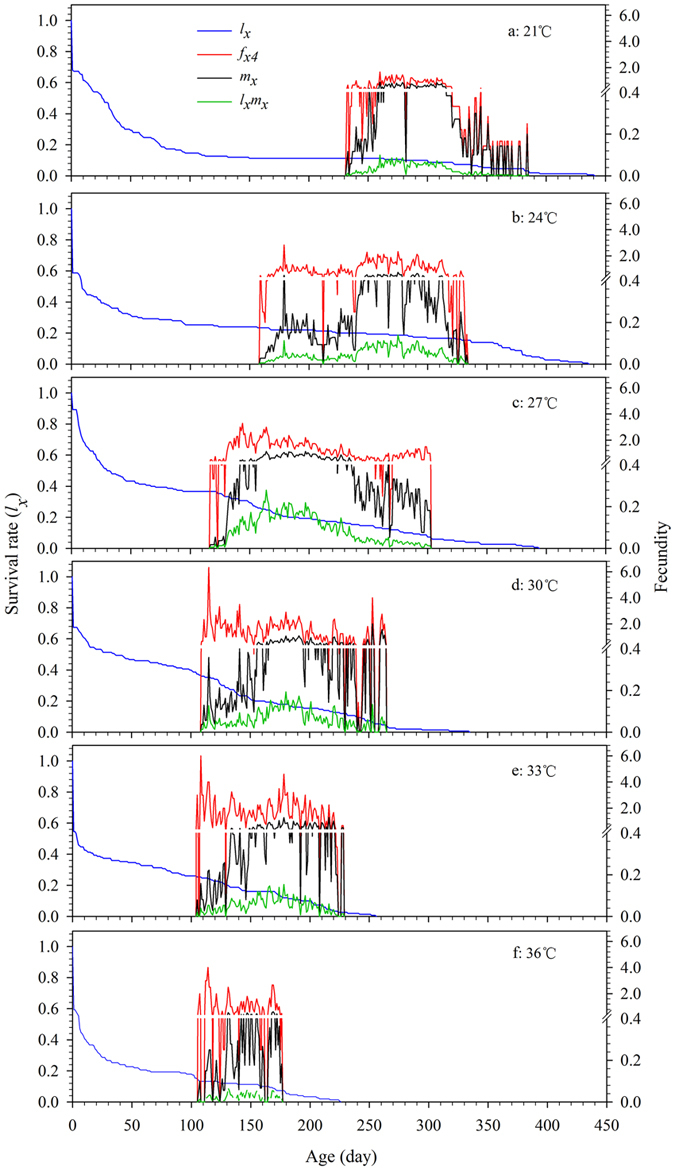
Age-specific survival rate (*l*_*x*_), female age-specific fecundity ( *f*_*x*4_), age-specific fecundity (*m*_*x*_), and age-specific maternity (*l*_*x*_*m*_*x*_) of *R. ferrugineus* reared on sugarcane at different constant temperatures. *f*_*x*4_, the mean fecundity of females at age *x* and stage 4 (female adult stage); *l*_*x*_, the probability of a newly laid egg surviving to age *x; m*_*x*_, the mean fecundity of individuals at age *x*.

The number of offspring produced by an individual weevil of age *x* and stage *j* is shown in Fig. 2a–f. In this species, females produce eggs and therefore there is only a single curve ( *f*_*x*4_) representing the females (stage 4). Different dynamic patterns for *f*_*x*4_, *m*_*x*_, and *l*_*x*_*m*_*x*_ were observed at the six temperatures. The starting time of reproduction ( *f*_*x*4_, *m*_*x*_, and *l*_*x*_*m*_*x*_) was earlier with increasing temperature: 233 d at 21 °C, but 105 and 106 d at 33 and 36 °C, respectively. A similar advancing trend was observed for the timing of the first reproductive peak (Fig. 2a–f). The *f*_*x*4_ peaks appeared at 260, 179, 144, 115, 108, and 114 d at 21, 24, 27, 30, 33 and 36 °C, respectively. The curve for *m*_*x*_ was lower than that for *f*_*x*4_ because it is a parameter of age-specific averaged fecundity that takes into account concurrent stages (Fig. 2a–f). The ranges of *f*_*x*4_*, m*_*x*_, and *l*_*x*_*m*_*x*_ were largest at 186 d at 27 °C and shortest at 71 d at 36 °C (Fig. 2c,f). Thus, the egg laying performance of *R. ferrugineus* females was more stable and durable at 27 °C.

Ignoring stage differentiation, the single age-specific survival rate (*l*_*x*_) gives the probability that an egg will survive to age *x*. We found that the curve of *l*_*x*_ fell slowest at 24 °C with 16.7% of individuals surviving longer than 300 d (Fig. 2b); *l*_*x*_ was relatively constant at 21 and 27 °C with 10.0% and 7.3% of individuals surviving longer than 300 d (Fig. 2a,c). However, there was a sharp fall in age-specific survival rate at 36 °C with no individual surviving longer than 250 d (Fig. 2f).

### Development, longevity, and fecundity

The durations of developmental stages varied significantly at the different temperatures throughout the life cycle (Table 1). The duration of the egg stage steadily decreased from 6.6 d at 21 °C to 2.2 d at 36 °C (*P* < 0.05), suggesting that it was sensitive to temperature variation (Table 1). The pre-adult stage did not differ in duration between 21 and 24 °C (*P* > 0.05), but decreased significantly at 27 and 30 °C (*P* < 0.05). However, no further significant decrease was observed above 30 °C (*P* > 0.05) (Table 1).

Longevity of adult females varied significantly at different temperatures (Table 1). The maximum longevity, 144.5 ± 10.8 d, was observed at 21 °C, but decreased at higher temperatures and fell to a minimum of 62.2 ± 14.4 d at 36 °C (*P* < 0.05) (Table 1). However, longevity in adult males showed a different response to temperature variation compared to female adults (Table 1). The maximum longevity occurred at 24 °C, which was significantly greater than that at 21 °C (*P* < 0.05), but did not differ significantly from that at 27 °C (*P* > 0.05). At temperatures above 27 °C, male longevity decreased significantly (Table 1). These changes suggest that males may be more sensitive to temperature variation.

The longest adult preoviposition period (APOP) occurred at 21 °C, while the shortest occurred at 27 °C and was only 4.7 ± 0.5 d; this period was significantly shorter than at 21, 24, and 30 °C (*P* < 0.05), but was similar to that at 33 and 36 °C (*P* > 0.05) (Table 1). The duration of the total preoviposition period (TPOP) fell from 249.7 ± 3.8 d at 21 °C to 125.0 ± 6.0 d at 36 °C (*P* < 0.05), similarly to the changes seen for pre-adult stage duration (Table 1). High temperatures greatly reduced the preoviposition period, suggesting an energy trade-off in the extreme environment.

The longest reproductive period was at 24 °C, but there was no significant difference across the range 21 to 30 °C (*P* > 0.05); the minimum period was observed at 36 °C (*P* < 0.05) (Table 1). Female fecundity initially increased as temperatures rose but then decreased at the highest temperatures. The highest female fecundity (125.0 ± 15.4) was observed at 27 °C, but did not vary significantly across the temperature range of 24 to 33 °C (*P* > 0.05) (Table 1). There was a sharp decline in fecundity at 36 °C with a mean of only 42.1 offspring, which was significantly lower than that at all other temperatures (*P* < 0.05).

### Age stage-specific life expectancy

**Figure 3:**
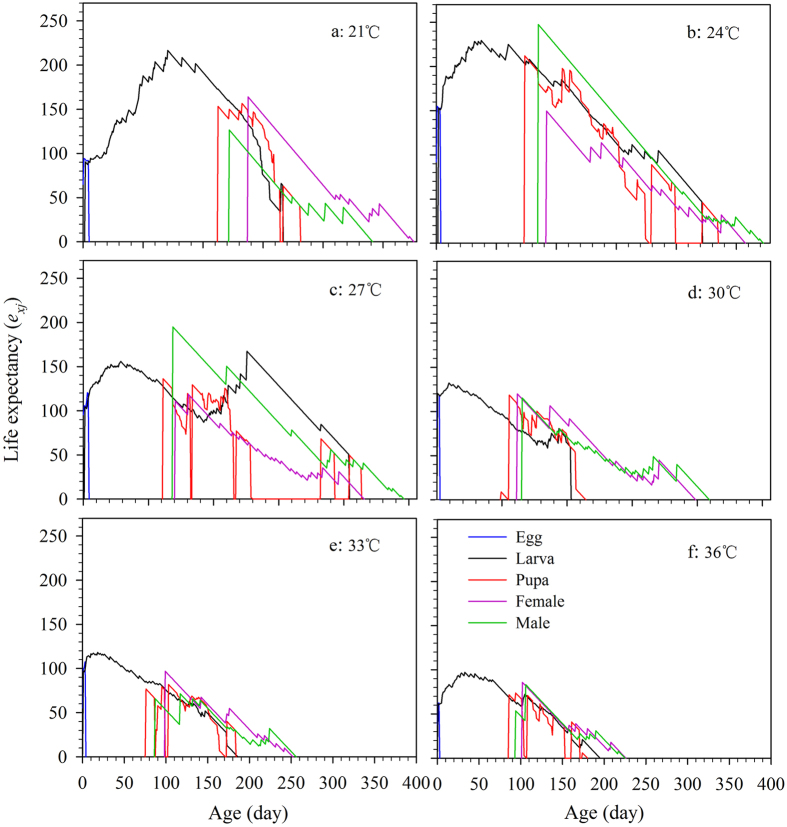
Age-stage- and sex-specific life expectancy (*e*_*xj*_) of *R. ferrugineus* reared on sugarcane at different constant temperatures. *e*_*xj*_, the future expected life span of an individual at age *x* and stage *j*.

The age stage-specific life expectancy (*e*_*xj*_) describes the future expected life span of an individual of age *x* and stage *j* (Fig. 3a–f). The life expectancies of newborn weevils (*e*_01_) were 90.4, 153.0, 104.4, 120.4, 97.2, and 61.6 d at 21, 24, 27, 30, 33, and 36 °C, respectively (Fig. 3a–f), showing that both low and high temperatures could shorten life expectancies. The *e*_*x*4_ of females fell from 164.0 d at 21 °C to 85.8 at 36 °C (Fig. 3a–f), while the maximum *e*_*x*4_ of males, 247.6 d, was observed at 24 °C (Fig. 3b), but decreased at higher temperatures and fell to a minimum of 53.6 d at 36 °C (Fig. 3f).

### Age stage-specific reproductive value

**Figure 4:**
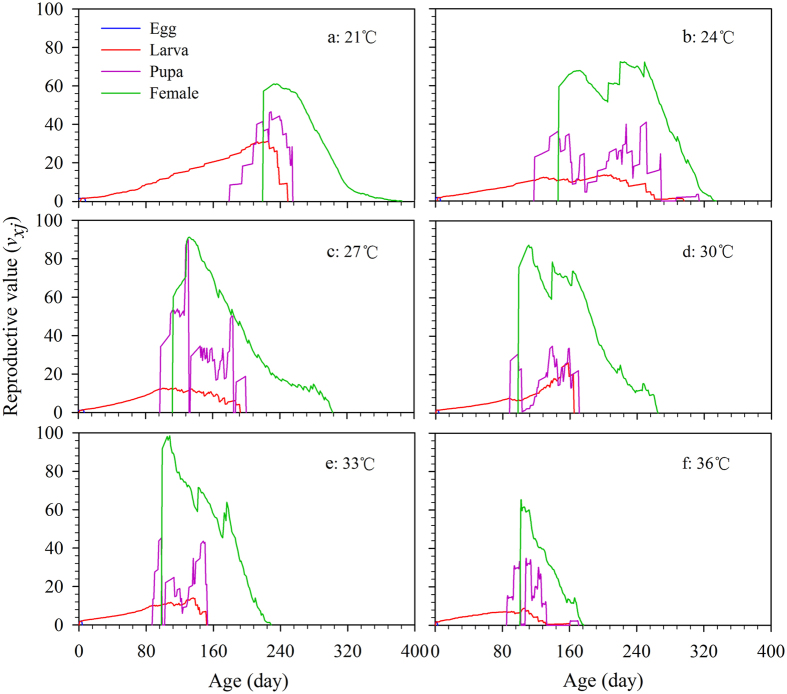
Age-stage reproductive value (*v*_*xj*_) of *R. ferrugineus* reared on sugarcane at different constant temperatures. *v*_*xj*_, the contribution of individuals at age *x* and stage *j* to the future population quantity.

The reproductive value (*v*_*xj*_) is the contribution of individuals of age *x* and stage *j* to the future population (Fig. 4a–f). After emergence of adult females at 220, 147, 113, 99, 99, and 102 d under temperature conditions from 21 to 36 °C, the *v*_*xj*_ jumped to 57.3, 59.5, 60.1, 75.9, 91.6, and 60.8 eggs, respectively with increasing temperature, while the peak *v*_*xj*_ occurred at 232 d (61.0 eggs), 220 d (72.5 eggs), 132 d (91.3 eggs), 111 d (87.4 eggs), 108 d (98.5 eggs), and 106 d (61.7 eggs) at the different temperatures (Fig. 4a–f). The longest duration of *v*_*x*4_ of female adults was 190 d at 27 °C, whereas it was only 75 d at 36 °C.

### Population parameters

All three parameters showed an initial increase and a maximum at 27 °C with 0.0152 d^−1^ for *r*, 1.0153 d^−1^ for *λ*, and 16.67 offspring for *R*_0_; they then fell significantly to 0.0027 d^−1^ for *r*, 1.0027 d^−1^ for *λ*, and 1.69 offspring for *R*_0_ at 36 °C, which were similar to those at 21 °C (*P* > 0.05) (Table 2). This suggests that the optimum temperature for *R. ferrugineus* population among those tested was 27 °C, and that low or high temperatures had a clear negative effect on population growth. On the other hand, the mean generation time (*T*) was significantly shortened as temperatures increased (Table 2).

### Population projection

**Figure 5:**
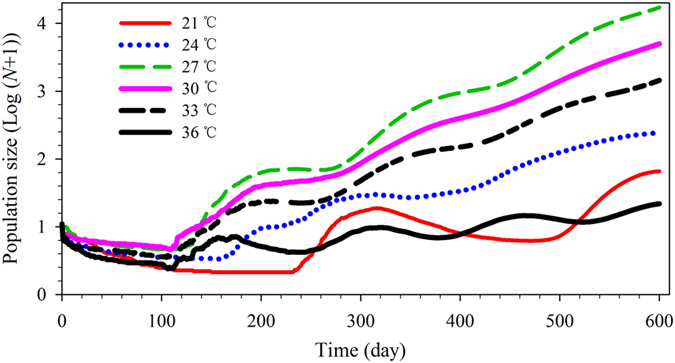
Population projection (total population size) of *R. ferrugineus* reared on sugarcane at different constant temperatures. Population projection started with 10 eggs of *R. ferrugineus.* The total population quantities of *R. ferrugineus* were assessed at different times, which reflected population growth potential under different constant temperatures.

**Figure 6:**
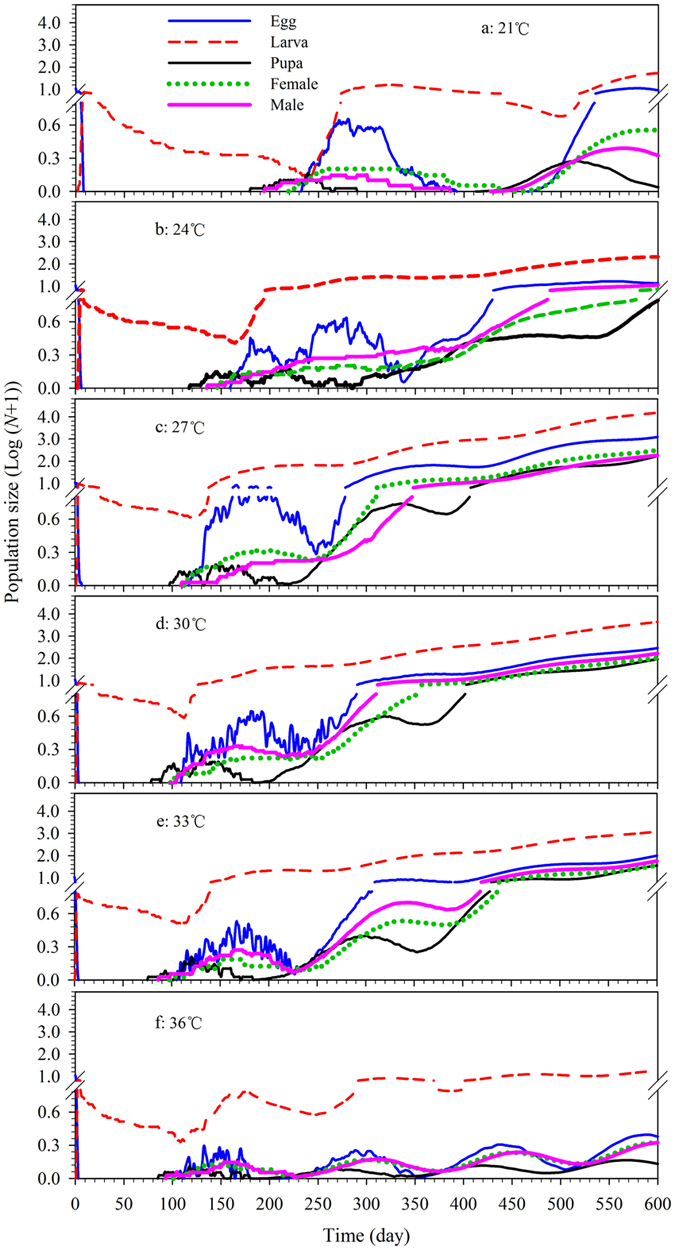
Population projection (population stage size) of *R. ferrugineus* reared on sugarcane at different constant temperatures. Population projection started with 10 eggs of *R. ferrugineus.* The population dynamics of *R. ferrugineus* in different stages were assessed at different times, which reflected the stable stage distribution under different constant temperatures.

From an initial 10 eggs, the fastest growing population was that at 27 °C; this population was predicted to exceed 17,057 individuals after 600 d (Fig. 5). Population size increases were slowest at 21 and 36 °C, with a final size estimate of 65 and 21 individuals after 600 d, respectively (Fig. 5). At 27, 30, and 33 °C, population growth followed a straight line after 350, 400, and 430 d, respectively, showing that these populations were approaching a stable stage distribution at the three temperatures (Fig. 6).

## Discussion

In ectothermic organisms such as insects, temperature is one of the most important environmental factors that regulate survival, development, reproduction, and seasonal occurrence^24^,^25^,^26^. Insects have an optimal temperature range for population growth and show significant restrictions on development at temperatures above or below the preferred range^27^,^28^. This temperature response has been found here in *R. ferrugineus*, where we found that *R. ferrugineus* populations survived under constant temperature conditions in the range 21 to 36 °C. The optimal temperature was 27 °C, which is consistent with the principal distributions of *R. ferrugineus* in China. We also found that *R. ferrugineus* was unable to complete development at 15 and 18 °C; this observation is consistent with the report by Li *et al.*^14^ that this species cannot complete development and reproduction at 16 °C (or 40 °C) on sugarcane and that the threshold temperature for egg hatching is 18.28 °C. Similarly, Zhao and Ju^29^ reported a survival rate for the generation of *R. ferrugineus* of only 10.0% at 19 °C. However, there are some contradictory data from other studies: Dembilio & Jacas^5^ and Dembilio *et al.*^12^ found that the threshold temperature for egg hatching was less than 14 °C on apple slices. These apparently contradictory differences may be attributable to geographical conditions, host plants, or other factors^26^,^30^.

In the present study, the durations of the immature stages of *R. ferrugineus* were temperature-dependent: the pre-adult stage fell from 237.8 ± 5.9 d to 131.8 ± 6.5 d as the temperature increased from 21 to 36 °C. These results are similar to those reported by Li *et al.*^14^ and Al-Nujiban *et al.*^31^, but are higher than the estimate obtained by Zhao & Ju^29^. These differences may be attributable to the different host plants^31^,^32^,^33^. Many studies have confirmed that the longest period for adult emergence occurs on sugarcane compared to date palm cultivars^31^,^33^. In addition, the prolongation of development times with decreasing temperatures may be due to the reduction in insect metabolism at lower temperatures, which has been reported for many insects including *Octodonta nipae*^18^, *Lemnia biplagiata*^34^, *Bradysia odoriphaga*^24^, and *Corythucha ciliata*^35^.

In insects, temperature is one of the most important determinants of reproduction to maintain populations^36^. This is especially the case for insect species that typically produce most offspring at an early age and have no parental care, such as *Callosobruchus chinensis*^37^, *Cylas formicarius elegantulus*^38^, and *Drosophila melanogaster*^39^. Our study showed the highest level of female fecundity was observed at 27 °C; fecundity at this temperature was significantly higher than at 21 and 36 °C. This trend in temperature-dependent fecundity has already been reported in *R. ferrugineus*^14^,^29^, and has also been found in *Ophraella communa*^27^, *Anabrus simplex*^40^, and *Bradysia odoriphaga*^24^. Some researchers argue that lower metabolic efficiency and more rapid energy consumption at low and high temperatures lead to a lower energy allocation to reproduction^41^. Thus, to ensure high-quality egg production, insects likely need to reduce their fecundity due to physiological trade-offs^42^. Fand *et al.*^26^ also reported reduced longevities in adults at high temperatures and a consequent shortening of the reproductive phase with decreased oviposition. Additionally, temperatures deviating from the normal range can alter reproductive physiology causing anomalous gonad development^43^, endocrine dyscrasia^44^, and abnormal sperm maturation and transfer^45^.

In general, the longevity of adult *R. ferrugineus* decreased with increasing temperature. This observation is consistent with the report by Zhao and Ju^29^ and in other insect species. We found a maximum female longevity at 21 °C and a decline to a minimum at 36 °C. Male longevity also sharply declined at 36 °C and was lower than that of female longevity. Sexual dimorphism in lifespan might result from sex-specific selection due to fundamental differences in how males and females optimize their fitness by allocating resources into current and future reproduction^45^,^46^. The reproductive strategies of males are more complicated than those of females and may involve higher energy requirements for successful mating (e.g., search for mates, competition, and courtship). As a result, limited energy resource availability at higher temperatures may make it necessary to sacrifice longevity for successful mating behaviour in males^45^,^46^. Liao *et al.*^47^ suggested that high temperatures suppress mating frequency and sperm transfer, indicating that substantial energy is required by the male for successful fecundity, resulting in an increased risk of death. In addition, many studies, especially in *Drosophila*, have found that the expression of genes affecting adult lifespan is temperature-dependent and that their relative sensitivity differs between the sexes^48^.

The curves for *s*_*xj*_, *f*_*xj*_, *e*_*xj*_, and *v*_*xj*_, which take into account the variable development rates, provide a comprehensive reflection of population dynamics^21^. Our results showed the occurrence time of the maximum survive rate of *R. ferrugineus* in the specific stage and the fecundity reach to the maximum at a specific age under different temperature regimes (Figs 1 and 2). These data will be of value for choosing the optimum time and strategy for pest control^49^,^50^. In addition, we found that both low and high temperatures have negative effects on the contribution of individuals of *R. ferrugineus* to the future population (Figs 3 and 4); the life expectancies of newborn weevils (*e*_01_) were relatively low at 21 and 36 °C, respectively (Fig. 3a–f). Similarly, the peak of *v*_*xj*_ was lowest (61.0 eggs) and occurred latest (232 d) at 21 °C, however, the peak value came earlier as temperatures increasing, but fell to a minimum of 61.7 eggs at 36 °C; this tendency is consistent with the report by Li *et al.*^25^, which is an important feature for evaluating the population growth potential^20^,^21^.

In this study, the intrinsic rate of increase (*r*), the finite rate of increase (*λ*), and the net reproductive rate (*R*_0_) of *R. ferrugineus* were at their highest at 27 °C (Table 2), which indicate that *R. ferrugineus* numbers may increase most rapidly at 27 °C. Interestingly, the emergence peaks of *R. ferrugineus* occur from May to June and from September to October in Fuzhou (Fujian), where the experimental population was initially collected, when the average temperature is about 27 °C; thus, our results may provide an important reference for risk assessment and management of *R. ferrugineus*. However, the population parameters were slightly lower than those reported by Li *et al.*^14^ and Zhao & Ju^29^ at the corresponding temperature, which might be attributable to differences in nutritional conditions or geographical populations^26^,^30^.

The fastest growing *R. ferrugineus* populations appeared at 27 and 30 °C, which may explain the suitability of Fujian province, and possibly the whole of subtropical and tropical zones, for *R. ferrugineus* (Fig. 5). In addition, change of stage structure during population growth can also be identified by population projection; stage structure is important for pest management because the dispersal and damage capability of the insects vary with stage^21^,^25^. As shown in Fig. 6, the *R. ferrugineus* populations reach the “stable age” or “stable age-stage” distribution after 350, 400, and 430 d, at 27, 30, and 33 °C, respectively. Furthermore, population projection offers a comprehensive understanding of the age and stage composition of a population during its growth. A population projection based on an age-stage, two-sex life table offers a comprehensive understanding of the age and stage composition of a population during its growth^21^,^25^.

In summary, we found that the growth rate and potential of *R. ferrugineus* populations was highest at 27 °C in an artificial environment. Using the age- and stage-specific life table approach, our study firstly simulated the population dynamics and evaluated the population growth potential of *R. ferrugineus* under different temperatures; this provide a vital foundation for determining the correct timing and strategies of chemical and biological control activities^51^, which often target pests at a specific age-stage, and for carrying out risk assessments. Here, we performed an experiment based only on the effects of temperature, a key abiotic factor for survival, development, and reproduction. Other environmental factors such as humidity, light, and rainfall that can influence pest population sizes were not considered. Humidity, for example, is especially important for rain-driven pests like *Apolygus lucorum*^52^ and *Lygus lineolaris*^53^. Although we speculate that temperature is the dominant abiotic factor affecting *R. ferrugineus*, an overall analysis of *R. ferrugineus* population dynamics needs to take a more full consideration of other environmental effects. Therefore, further studies are required to extend our results either by adding different abiotic factors or even applying abiotic-biotic interactions for *R. ferrugineus* based on temperature-dependent phenology.

## Materials and Methods

### Insect rearing

The *R. ferrugineus* adults used here were derived from insects collected in 2008 from a Canary Island date palm (*Phoenix canariensis* Hort. ex Chabaud) on the campus of Fujian Agriculture and Forestry University (FAFU), Fujian, China. A colony of the red palm weevils was maintained at 25 ± 0.5 °C and 75 ± 5% relative humidity with a 12 h light/ 12 h dark schedule in a growth chamber (PRX-250B-30, Haishu Saifu Experimental Instrument Factory, Ningbo, China) in our laboratory. As described previously^54^, the weevils were reared on fresh sugarcane stem tissues in clean plastic bottles (70 mm diameter, 105 mm height; Jiafeng Horticultural Products Co. Ltd., Shanghai, China) with moist filter paper to maintain humidity. The bottle neck was covered with fine mesh gauze to allow air ventilation. All sugarcane materials were bought from the fruit shop on campus. Every 2 d, the bottles were cleaned and fresh sugarcanes were added as necessary. To obtain a large breeding population, five pairs of *R. ferrugineus* adults per bottle and a total of 100 bottles were used to initiate the colony, and insects from the field populations were added to the laboratory colony every six months to maintain high genetic diversity. After two generations, the offspring were used for experimental studies.

### Constant temperature experiment

The effects of different temperatures on population growth were tested by collecting newly-laid eggs from the laboratory colony and incubating them on petri dishes in growth chambers running at eight different temperatures (15, 18, 21, 24, 27, 30, 33, and 36 ± 0.5 °C); humidity and day length conditions were as described above. Fifty eggs, laid within a 24 h period, were placed on filter paper in a Petri dish (9 cm diameter) with a moist cotton wick to maintain humidity. Each petri dish was considered as one replicate, and three replicates were used at each temperature. The development time and hatching rate of eggs at each temperature were recorded and the moist cotton wick was changed daily. Each hatched larva was transferred individually to a new plastic bottle as described above. Fresh sugarcanes cut into small pieces (35 mm × 35 mm × 20 mm) with knife were provided as food and changed every two days until the larvae pupated. The moist filter paper was checked daily and renewed as needed. Development time and survival individual number were recorded daily. Pupae were collected and kept individually in plastic bottles for emergence and sex determination.

After emergence, male and female pairs were placed in individual plastic bottles with a piece of sugarcane as food and egg laying substrate. The sugarcane was also changed every two days. The number of eggs laid was monitored daily and the longevity of the adults was recorded.

### Statistical analysis

The data from the different temperatures were analysed using an age-stage and two-sex life table approach^23^. The life history parameters, including age stage-specific survival rate (*s*_*xj*_, the probability that a newly laid egg will survive to age *x* and stage *j*), age stage-specific fecundity (*f*_*x*4_, the mean fecundity of females at age *x*), age-specific survival rates (*l*_*x*_, the probability of a newly laid egg surviving to age *x*), age-specific fecundity (*m*_*x*_, the mean fecundity of individuals at age *x*), age-specific maternity (*l*_*x*_*m*_*x*_), age-stage life expectancy (*e*_*xj*_), age-stage reproductive value (*v*_*xj*_) and the demographic parameters of intrinsic rate of increase (*r*), finite rate of increase (λ), net reproductive rate (*R*_0_), mean generation time (*T*) were estimated and calculated using the computer program TWOSEX-MSChart^55^ (http://140.20.197.173/Ecology/Download/Twosex-MSChart.rar, last accessed 25 June 2015), which is designed in Visual BASIC for the Windows operating system and is available at http://nhsbig.inhs.uiuc.edu/wes/chi.html (Illinois Natural History Survey, Champaign-Urbana, IL). For population demographic variables, the bootstrap technique included in the TWOSEX-MSChart programme was used to estimate the means and standard errors for development time, longevity, adult preoviposition period (APOP), total preoviposition period (TPOP), oviposition days, fecundity, and the population parameters (*r, λ, R*_0_, and *T*)^21^,^56^,^57^ with 100,000 bootstrap replicates (*B* = 100,000). Differences among the different temperatures were compared by paired bootstrap tests based on the confidence interval of the difference between means^21^,^56^,^57^.

Projections of population growth of *R. ferrugineus* at different temperatures were based on the age-stage, two-sex life table theory^23^,^51^ and obtained using the TIMING-MSChart programme^55^. For comparison, an initial population of 10 eggs was used for the simulation at each temperature.

## Additional Information

**How to cite this article**: Peng, L. *et al*. Demographic comparison and population projection of *Rhynchophorus ferrugineus* (Coleoptera: Curculionidae) reared on sugarcane at different temperatures. *Sci. Rep.* **6**, 31659; doi: 10.1038/srep31659 (2016).

## Figures and Tables

**Table 1:** Means and standard errors of development time, adult longevity, adult preoviposition period (APOP), total preoviposition period (TPOP), oviposition days, fecundity of *R. ferrugineus* at six constant temperatures.

Stage	Temperature (°C)					
	21	24	27	30	33	36
Egg (d)	6.6 ± 0.1 a	4.4 ± 0.1 b	3.3 ± 0.1 c	2.7 ± 0.1 d	2.3 ± 0.1 e	2.2 ± 0.1 f
Larva (d)	215.4 ± 5.6 a	197.7 ± 9.8 a	144.1 ± 8.1 b	120.2 ± 3.5 c	120.1 ± 6.3 c	122.7 ± 5.8 c
Pupa (d)	16.1 ± 0.8 b	19.3 ± 0.8 a	12.3 ± 0.3 c	11.9 ± 0.3 c	10.7 ± 0.4 d	8.8 ± 0.3 e
Preadult (d)	237.8 ± 5.9 a	218.2 ± 10.7 a	165.1 ± 8.4 b	135.3 ± 3.7 c	134.9 ± 4.4 c	131.8 ± 6.5 c
Female adult (d)	144.5 ± 10.8 a	112.0 ± 18.4 ab	94.7 ± 10.1 bcd	99.4 ± 12.6 bc	69.0 ± 10.3 cd	62.2 ± 14.1 d
Male adult (d)	87.1 ± 13.8 b	158.0 ± 11.7 a	132.2 ± 15.9 a	89.5 ± 9.6 b	56.3 ± 7.0 c	45.9 ± 12.2 c
APOP (d)	10.1 ± 1.0 a	8.2 ± 1.2 ab	4.7 ± 0.5 d	7.0 ± 1.0 bc	6.1 ± 0.5 bcd	4.8 ± 0.9 cd
TPOP (d)	249.7 ± 3.8 a	204.9 ± 13.5 b	151.5 ± 5.4 c	144.3 ± 7.5 c	139.0 ± 6.7 cd	125.0 ± 6.0 d
Oviposition days	53.6 ± 4.1 a	59.9 ± 11.4 a	56.2 ± 6.3 a	48.0 ± 5.7 ab	31.8 ± 6.9 bc	23.6 ± 3.2 c
Fecundity (egg)	75.2 ± 7.9 bc	97.0 ± 21.1 ab	125.0 ± 15.4 a	124.1 ± 17.9 a	108.1 ± 25.9 ab	42.1 ± 10.3 d

Means in the same row followed by the same letter are not significantly different. The SEs were estimated using 100,000 bootstraps and compared using a paired bootstrap test based on CI of differences.

**Table 2:** Means and standard errors of the intrinsic rate of increase (*r*), finite rate of increase (λ), net reproductive rate (*R*_0_), mean generation time (*T* ) of *R. ferrugineus* at six constant temperatures.

Population parameters	Temperature (°C)					
	21	24	27	30	33	36
*r* (d^−1^)	0.0050 ± 0.0013 c	0.0083 ± 0.0015 bc	0.0152 ± 0.0015 a	0.0128 ± 0.0021 ab	0.0109 ± 0.0030 abc	0.0027 ± 0.0037 c
λ (d^−1^)	1.0050 ± 0.0013 c	1.0084 ± 0.0016 bc	1.0153 ± 0.0015 a	1.0129 ± 0.0021 ab	1.0109 ± 0.0031 abc	1.0027 ± 0.0037 c
*R*_0_ (offspring/individual)	4.51 ± 1.52 bc	8.41 ± 2.84 ab	16.67 ± 4.02 a	9.93 ± 3.08 ab	6.49 ± 2.56 bc	1.69 ± 0.76 c
*T* (d)	287.90 ± 5.32 a	248.99 ± 9.89 b	183.30 ± 6.76 c	176.16 ± 9.62 c	164.08 ± 9.85 cd	144.89 ± 7.57 d

Means in the same row followed by the same letter are not significantly different. The SEs were estimated using 100,000 bootstraps and compared using a paired bootstrap test based on the CI of difference.
